# Construction of an RNA-Binding Protein-Related Prognostic Model for Pancreatic Adenocarcinoma Based on TCGA and GTEx Databases

**DOI:** 10.3389/fgene.2020.610350

**Published:** 2021-01-27

**Authors:** Xin Wen, Zhiying Shao, Shuyi Chen, Wei Wang, Yan Wang, Jinghua Jiang, Qinggong Ma, Longzhen Zhang

**Affiliations:** ^1^Department of Radiation Oncology, Affiliated Hospital of Xuzhou Medical University, Xuzhou, China; ^2^Department of Interventional Ultrasound, Cancer Hospital of the University of Chinese Academy of Sciences (Zhejiang Cancer Hospital), Hangzhou, China; ^3^Institute of Cancer and Basic Medicine (IBMC), Chinese Academy of Sciences, Hangzhou, China; ^4^Cancer Institute, Xuzhou Medical University, Xuzhou, China; ^5^Jiangsu Center for the Collaboration and Innovation of Cancer Biotherapy, Xuzhou, China

**Keywords:** RNA-binding proteins (RBPs), pancreatic adenocarcinoma (PAAD), prognosis, TCGA, GTEx

## Abstract

**Background:** Recently, RNA-binding proteins (RBPs) were reported to interact with target mRNA to regulate gene posttranscriptional expression, and RBP-mediated RNA modification can regulate the expression and function of proto-oncogenes and tumor suppressor genes. We systematically analyzed the expression of RBPs in pancreatic adenocarcinoma (PAAD) and constructed an RBP-associated prognostic risk model.

**Methods:** Gene expression data of normal pancreatic samples as well as PAAD samples were downloaded from TCGA-PAAD and GTEx databases. Wilcoxon test and univariate Cox analysis were, respectively, applied to screen differential expression RBPs (DE-RBPs) and prognostic-associated RBPs (pRBPs). Functional enrichment was analyzed by GO, KEGG, and GSEA. Protein–protein interaction (PPI) network was constructed by STRING online database. Modeling RBPs were selected by multivariate Cox analysis. Kaplan–Meier survival and Cox analysis were applied to evaluate the effects of risk score on the overall survival of PAAD patients. ROC curves and validation cohort were applied to verify the accuracy of the model. Nomogram was applied for predicting 1-, 3-, and 5-year overall survival (OS) of PAAD patients. At last, modeling RBPs were further analyzed to explore their differential expression, prognostic value, as well as enrichment pathways in PAAD.

**Results:** RBPs (453) were differentially expressed in normal and tumor samples, besides, 28 of which were prognostic associated. DE-RBPs (453) are functionally associated with ribosome, ribonuclease, spliceosome, etc. Eight RBPs (PABPC1, PRPF6, OAS1, RBM5, LSM12, IPO7, FXR1, and RBM6) were identified to construct a prognostic risk model. Higher risk score not only predicted poor prognosis but also was an independent poor prognostic indicator, which was verified by ROC curves and validation cohort. Eight modeling RBPs were confirmed to be significantly differentially expressed between normal and tumor samples from RNA and protein level. Besides, all of eight RBPs were related with overall survival of PAAD patients.

**Conclusions:** We successfully constructed an RBP-associated prognostic risk model in PAAD, which has a potential clinical application prospect.

## Introduction

Pancreatic adenocarcinoma (PAAD) is one of the malignant tumors with the worst prognosis in the digestive system. On the background of great advances in diagnosis and therapy of PAAD, there are only 2–9% of PAAD patients surviving for more than 5 years, indicating that PAAD is a highly fatal disease with an insidious onset (McGuigan et al., 2018). Existing medical technologies such as imaging examination, tumor molecular biomarkers, and pathological examination are currently quite limited and in low efficiency, which may be a momentous reason for low early detection rate and high mortality in PAAD. Therefore, exploring the pathogenesis of PAAD, formulating effective methods of early screening and diagnosis, and looking for new prognostic biomarkers and treatment targets of PAAD are helpful to improve the therapeutic effect and survival rate of PAAD.

RNA-binding proteins (RBPs) are defined as proteins that contain known domains to directly interact with various types of RNA. Of course, some proteins without structurally characterized conformations to interact with RNA but residing within well characterized ribonucleoproteins (RNPs–protein or proteins complexed with RNA as an obligate binding partner) are also defined as RBPs. With the development of techniques for the identification of RBPs, 1,542 RBPs have been in a final census up to date (Gerstberger et al., 2014). RBPs play a vital role in regulating post-transcription of gene expression by recognizing specific sequences or secondary structures to form ribonucleoprotein (RNP) complexes to regulate a series of RNA processes, including splicing, polyadenylation, maturation, modification, transport, stability, localization, and translation (Castello et al., 2012; Mitchell and Parker, 2014). RBPs is a key regulator in maintaining cell physiological balance, especially in stress response (such as hypoxia, DNA damage, nutrition deficiency, or chemotherapy) and cell development (Masuda and Kuwano, 2019). More studies have proved that abnormal RBP expression is the genesis of diseases and is associated with cancer occurring and development (Pereira et al., 2017; Chatterji and Rustgi, 2018; Legrand et al., 2019; Li et al., 2020). The role of RBP-promoters or suppressors are various according to cancer types. For example, RBM38 was considered to take part in the formation of T-cell lymphoma by regulating mutants p53 and PTEN, but in non-small cell lung cancer (NSCLC), renal carcinoma (RCC), and hepatocellular carcinoma (HCC), RBM38 suppressed the progression of carcinoma (Ding et al., 2014; Huang et al., 2017; Yang et al., 2018; Zhang et al., 2018). PCBP1 was found in a lower expression level in cervical, colorectal, lung, liver, and breast cancer compared with corresponding normal tissues, which was identified as a tumor suppressor (Pillai et al., 2003; Thakur et al., 2003; Wang et al., 2010; Zhang et al., 2010; Guo and Jia, 2018). Not only does RBPs itself influence occurrence and progression of cancers, RBPs can also influence the expression of other oncogenes and tumor suppressor genes through post-translational modification, expression, or localization, which regulates the growth of cancers (Lujan et al., 2018). At present, there is no systematic study to analyze the relationship of RBP expression with PAAD.

**FIGURE 1:**
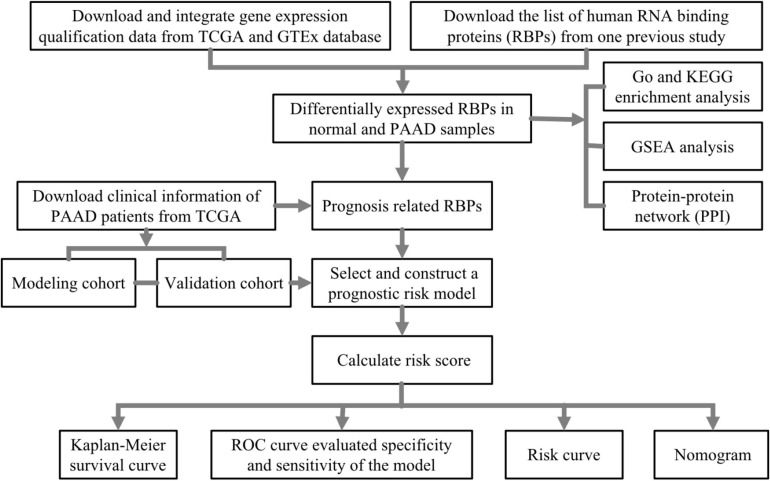
The flowchart of analysis of RNA-binding proteins (RBPs) in pancreatic adenocarcinoma (PAAD).

In this study, we first identified differently expressed and prognosis-related RBPs in PAAD through high-throughput bioinformatics analysis based on TCGA and GTEx database, and ultimately, we constructed and verified the prognostic risk model, which may become potential diagnostic and prognostic biomarkers. The complete workflow is summarized in Figure 1.

## Materials and Methods

### Data Download and Processing

Gene expression data of normal pancreatic samples and PAAD samples were downloaded from GTEx databases^1^ and TCGA-PAAD^2^. Data type was HTseq-FPKM, and gene expression level in both two databases was further processed by log_2_ (FPKM+1). It should be noted that GTEx database collected more than 7,000 normal samples from 449 healthy humans, and gene expression data were treated by the same sequencing platform with TCGA database for minimizing potential batch effects. Previous studies have proved that the gene expression data of TCGA and GTEx can be analyzed and integrated successfully (Kosti et al., 2016; Aran et al., 2017; Raphael et al., 2017; Zeng et al., 2019; Venkat et al., 2020). Based on this, we integrated gene expression data from TCGA-PAAD and GTEx including 178 tumor samples and 171 normal samples (4 from TCGA-PAAD and 167 from GTEx). Clinical information was all downloaded from TCGA-PAAD, and patients losing to follow-up or follow-up of less than 90 days were removed. Ultimately, 173 PAAD samples, summarized in Table 1, were brought into survival analysis. The list of 1,542 RBPs in a final census up to date was downloaded from one previous study of Gerstberger et al. (2014).

**TABLE 1:** Clinical characteristics of pancreatic adenocarcinoma (PAAD) patients from TCGA.

**Clinical characteristics**		**Total (173)**	**%**
Age at diagnosis (y)	<65	88	50.87
	≥65	85	49.13
Gender	Female	79	45.66
	Male	94	54.34
Fustat	Live	78	45.09
	Death	95	54.91
Grade	G1	29	16.76
	G2	93	53.76
	G3	46	26.59
	G4	2	1.16
	Gx	3	1.73
Clinical stage	Stage I	19	10.98
	Stage II	142	82.08
	Stage III	4	2.31
	Stage IV	5	2.89
	Unknown	3	1.73
T stage	T1	7	4.05
	T2	21	12.14
	T3	139	80.35
	T4	4	2.31
	Tx	2	1.16
Distant metastasis	Negative	79	45.66
	Positive	5	2.89
	Mx	89	51.45
Lymph nodes	Negative	48	27.75
	Positive	120	69.36
	Nx	5	2.89

### Variance Analysis

Differential expression-RBPs (DE-RBPs) in normal and PAAD samples were selected by Wilcoxon test with the screening requirement of false discovery rate (FDR) < 0.01 and | Log2FC| > 1 (Zhang et al., 2016; Han et al., 2018; Ouyang et al., 2019). In variance analysis, if one gene appears for more than one time, the mean value was calculated and applied for further analysis, which is by means of the “Limma” package (Li et al., 2020).

### Functional Enrichment Analysis and Protein–Protein Interaction Network

The GO and KEGG functional enrichment of DE-RBPs were analyzed with the requirement of *p*-value < 0.05 and *q*-value < 0.05 (Yang et al., 2019). Functional enrichment analysis of these 453 DE-RBPs was further analyzed by gene set enrichment analysis (GSEA). GSEA is a computational method, which determines whether an *a priori* defined set of genes shows significant differences between two groups (normal pancreatic group and PAAD group) statistically. One thousand genome permutations were performed per analysis (Subramanian et al., 2005; Wu and Zhang, 2018). | Normalized enrichment score| (| NES|) > 1 and norminal *p*-value (NOM *p*-value) < 0.05 were considered significant. Functional protein–protein interaction (PPI) network of DE-RBPs was analyzed by STRING online database^3^ (Szklarczyk et al., 2019) and was visualized by cytoscape 3.7.1 (Zhang et al., 2016). The important precondition was set as interaction score ≥ 0.4 and hiding disconnected nodes. Critical sub-networks were separated out to construct sub-networks by MCODE (molecular complex detection) plug-in of the cytoscape 3.7.1 software (Bader and Hogue, 2003). Node count number and MCODE score were both more than 15.

### Construction of Prognostic Risk Model in PAAD

Prognosis-related RBPs (pRBPs) were selected by univariate Cox analysis with the screening criteria of *p* ≤ 0.001, which was visualized with “forestplot” software package in R software (Li et al., 2020). pRBP constructing prognostic risk model in PAAD were further analyzed and selected by multivariate Cox analysis. Risk score was calculated by the formulation $\mathrm{Risk}\,\mathrm{Score}=\sum_{1}^{n}\text{coef}\times E\,\text{xp}\,n$, where coef is the coefficient value of one pRBP constructed model, Exp is the expression level of the corresponding gene, and n is the number of modeling pRBPs. Here, patients from TCGA-PAAD were classified into two cohorts randomly and equally. One cohort was the modeling, and the other was the testing. Based on the median risk scores of two cohorts, PAAD patients were split into two subgroups, respectively: the high-risk score subgroups and the low-risk score subgroups. Kaplan–Meier (KM) survival curves were used to describe the overall survival difference between two subgroups by the “survminer” package, and ROC (receiver operating characteristic) curves were used to evaluate the accuracy of the model with AUC values (area under curve) by “survivalROC” package (Heagerty et al., 2000). Cox regression analysis was further applied to analyze the relationship of risk score prognosis of PAAD patients. Finally, the “rms” package was used to draw a nomogram for predicting 1-, 3-, and 5-year OS of PAAD patients in modeling cohort (Kim et al., 2018; Liu et al., 2020).

### Differential Expression, Prognostic Analysis, and Gene Set Enrichment Analysis of 8 Modeling RBPs

Wilcox regression was applied to analyze the expression of modeling RBPs in normal samples and PAAD samples (Zhang et al., 2016; Han et al., 2018; Ouyang et al., 2019). A *P*-value < 0.05 was considered significant. The differential expression of eight modeling RBPs in normal and tumor cases was then verified by the Human Protein Atlas (HPA) online database^4^ from protein level in forms of immunohistochemical staining images (Thul et al., 2017). Kaplan–Meier survival curves were applied to analyze prognostic values of modeling RBPs. A *P*-value < 0.05 was considered significant. We further analyzed the function and potential molecular mechanism of eight modeling RBPs by GSEA. GSEA was conducted as abovementioned and in a previous study (Zhang et al., 2020).

### Statistical Analysis

All statistical analyses in this study were performed by the R (V.4.0.2) software.

## Results

### Patient Characteristics

As summarized in Table 1, basic information of 173 PAAD patients downloaded from TCGA database on June 11, 2020 were used for analysis. Patients older than 65 and younger than 65 accounted for half approximately, respectively, and male patients were approximately 8.68% more than female patients. Until the date we downloaded information, 54.91% PAAD patients have died. Of the 173 PAAD patients, the pathologic stage mainly focused on the G2 stage (*n* = 93, 53.76%) and G3 stage (*n* = 46, 26.59%); the clinical stage mainly concentrated on stage II (*n* = 142, 82.08%). Stages I, III, and IV patients accounted for 10.98% (*n* = 19), 2.31% (*n* = 4), and 2.89% (*n* = 5), respectively. Patients with T3 stage accounted for the majority (*n* = 139, 80.35%). Tx, T1, T2, and T4 patients accounted for 1.16% (*n* = 2), 4.05% (*n* = 7), 12.14% (*n* = 21), and 2.31% (*n* = 4), respectively. Patients with positive lymph node metastasis and distant metastasis accounted for 69.36% (*n* = 120) and 2.89% (*n* = 5), respectively, which further proved that insidious onset of PAAD resulted in advanced stage and poor prognosis when diagnosed.

### Variance Analysis

**FIGURE 2:**
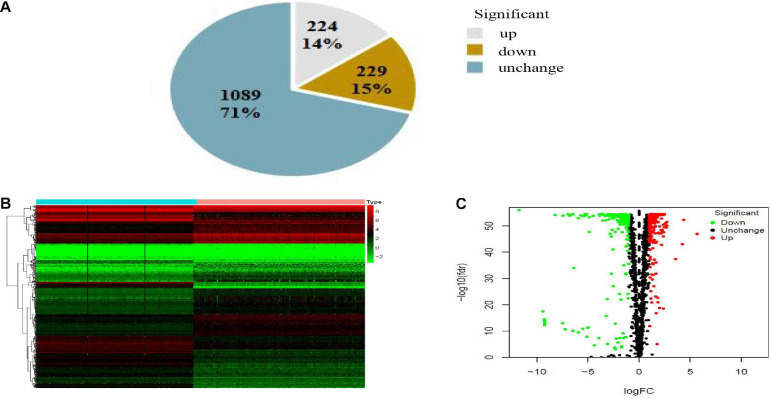
Pie chart **(A)**, heatmap **(B)**, and volcano map **(C)** of RBPs in PAAD. **(A)** Gray, brown, and blue pieces represented number and percentage of up-, down- and no significant difference RBPs. **(B)** *X*- and *Y*-axes represented samples and RBPs, respectively. Higher, lower, and medium expression of RBPs is shown, respectively, in red, green, and black. Blue and pink bars on top of the heat map indicate normal pancreatic samples and PAAD samples, respectively. **(C)** Downregulated, upregulated, and no significant difference RBPs in PAAD are shown in green, red, and black dots, respectively. False discovery rate (FDR) < 0.01 and | log_2_FC| > 1 was considered statistically significant.

The expression variance of 1,542 RBPs between 171 normal samples and 178 PAAD samples is shown in Figure 2. RBPs (453) were differentially expressed in normal and tumor samples (FDR < 0.01 and | log2FC| > 1). Compared with normal samples, 224 DE-RBPs were upregulated and 229 DE-RBPs were downregulated in PAAD samples.

### Functional Enrichment Analysis

**FIGURE 3:**
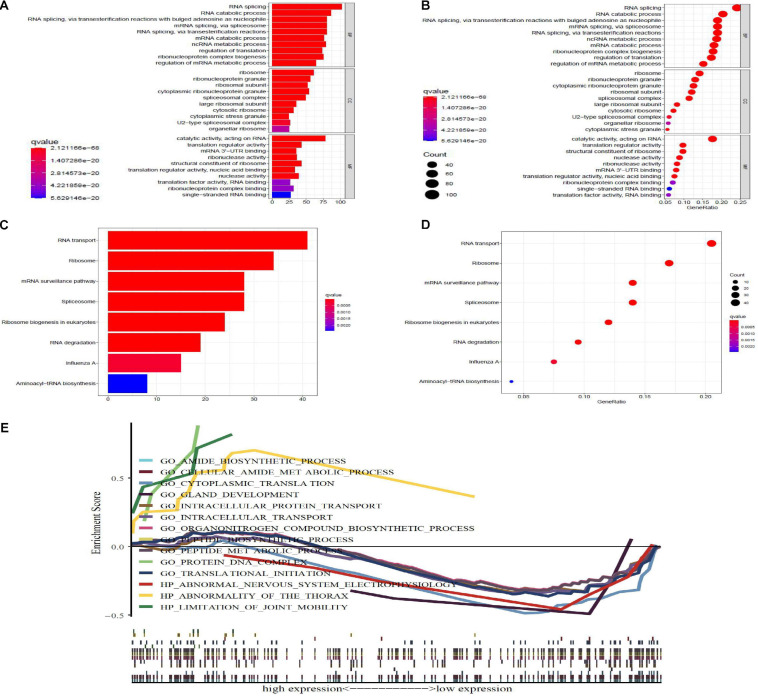
GO, KEGG, and gene set enrichment analysis (GSEA), enrichment analysis of differential expression-RBPs (DE-RBPs) in PAAD. Panels **(A,B)** are barplot and bubble plot, respectively, of GO functional enrichment analysis; BP, biological process; CC, cellular component; MF, molecular function. Panels **(B,D)** are barplot and bubble plot, respectively, of KEGG pathway enrichment analysis. *P*-value < 0.05 and *q*-value < 0.05 are considered statistically significant. **(E)** GSEA analysis for DE-RBPs. *P*-value < 0.05 and normalized enrichment score | NES| > 1 were considered statistically significant.

To research the function and molecular mechanisms of 453 DE-RBPs, we subsequently implemented GO functional, KEGG pathway enrichment analysis, and GSEA. GO functional enrichment analysis was classified by three categories: BP—biological process, CC—cellular component, and MF—molecular function. As shown in Table 2A and Figure 3, the top 10 components of BP were RNA splicing, RNA catabolic process, etc. The top 10 components of CC were ribosome, ribonucleoprotein granule, etc. The top 10 components of MF were catalytic activity acting on RNA, translation regulator activity, etc. According to the NES of GSEA, the function of DE-RBPs in PAAD were enriched in the protein DNA complex, abnormality of the thorax, and limitation of joint mobility; the function of DE-RBPs in normal pancreatic group were enriched in cytoplasmic translation, peptide metabolic process, peptide biosynthetic process, etc. (Supplementary Figure 1, Table 2B and Figure 3). The KEGG pathway enrichment analysis showed that DE-RBPs in PAAD were enriched in RNA transport and ribosome mainly. None enriched pathways were found by GSEA. Therefore, RBPs may mediate various regulatory processes of post-transcription, such as RNA splicing and polyadenylation, which then affect the occurrence and progression of malignant tumors and other biological functions.

**TABLE 2A:** GO and KEGG functional enrichment analysis of differential expression –RBPs (DE-RBPs) in PAAD.

**Classification**	**ID**	**Description**	***p*-value**	***q*-value**	**Count**
GO-BP	GO:0008380	RNA splicing	1.02E-71	2.12E-68	102
	GO:0006401	RNA catabolic process	6.83E-60	7.09E-57	86
	GO:0000377	RNA splicing, via transesterification reactions with bulged adenosine as nucleophile	1.15E-54	5.99E-52	80
	GO:0000398	mRNA splicing, via spliceosome	1.15E-54	5.99E-52	80
	GO:0000375	RNA splicing, via transesterification reactions	2.21E—54	9.18E-52	80
	GO:0006402	mRNA catabolic process	1.69E-51	5.84E-49	76
	GO:0034660	ncRNA metabolic process	9.76E-46	2.90E-43	79
	GO:0006417	Regulation of translation	2.85E-43	7.41E-41	73
	GO:0022613	Ribonucleoprotein complex biogenesis	3.41E-42	7.87E-40	75
	GO:1903311	Regulation of mRNA metabolic process	1.06E-41	2.21E-39	64
GO-CC	GO:0005840	Ribosome	5.46E-44	1.03E-41	61
	GO:0035770	Ribonucleoprotein granule	2.64E-43	2.49E-41	56
	GO:0044391	Ribosomal subunit	2.45E-42	1.54E-40	52
	GO:0036464	Cytoplasmic ribonucleoprotein granule	4.00E-42	1.88E-40	54
	GO:0005681	Spliceosomal complex	3.68E-39	1.39E-37	49
	GO:0015934	Large ribosomal subunit	1.13E-29	3.56E-28	35
	GO:0022626	Cytosolic ribosome	9.46E-26	2.55E-24	31
	GO:0010494	Cytoplasmic stress granule	2.01E-23	4.74E-22	24
	GO:0005684	U2-type spliceosomal complex	2.57E-22	5.38E-21	26
	GO:0000313	Organellar ribosome	1.93E-21	3.30E-20	25
GO-MF	GO:0140098	Catalytic activity, acting on RNA	2.03E-48	5.46E-46	78
	GO:0045182	Translation regulator activity	6.48E-35	8.70E-33	43
	GO:0003730	mRNA 3′-UTR binding	1.19E-32	1.07E-30	35
	GO:0004540	Ribonuclease activity	5.49E-30	3.68E-28	36
	GO:0003735	Structural constituent of ribosome	8.86E-28	4.75E-26	43
	GO:0090079	Translation regulator activity, nucleic acid binding	6.70E-27	3.00E-25	33
	GO:0004518	Nuclease activity	2.24E-23	8.60E-22	39
	GO:0008135	Translation factor activity, RNA binding	1.44E-21	4.31E-20	26
	GO:0043021	Ribonucleoprotein complex binding	1.44E-21	4.31E-20	31
	GO:0003727	Single-stranded RNA binding	2.10E-21	5.63E-20	27
KEGG	hsa03013	RNA transport	2.83E-28	2.59E-26	41
	hsa03010	Ribosome	4.98E-23	1.63E-21	34
	hsa03015	mRNA surveillance pathway	5.33E-23	1.63E-21	28
	hsa03040	Spliceosome	2.47E-17	5.64E-16	28
	hsa03008	Ribosome biogenesis in eukaryotes	1.59E-16	2.91E-15	24
	hsa03018	RNA degradation	3.30E-14	5.04E-13	19
	hsa05164	Influenza A	2.11E-05	2.77E-04	15
	hsa00970	Aminoacyl-tRNA biosynthesis	2.11E-04	2.42E-03	8

*BP, biological process; CC, cellular component; MF, molecular function. p-value < 0.05 and q-value < 0.05 was considered statistically significant.*

**TABLE 2B:** Functional enrichment analysis of DE-RBPs in PAAD by gene set enrichment analysis (GSEA).

	**Name**	**ES**	**NES**	***P*-value**
GSEA	GO protein DNA complex	0.88	1.48	0.00
	HP abnormality of the thorax	0.70	1.41	0.03
	HP limitation of joint mobility	0.82	1.35	0.03
	GO cytoplasmic translation	−0.51	−1.62	0.01
	GO peptide metabolic process	−0.33	−1.56	0.01
	GO peptide biosynthetic process	−0.33	−1.56	0.01
	GO cellular amide metabolic process	−0.33	−1.56	0.01
	GO amide biosynthetic process	−0.33	−1.56	0.01
	GO organonitrogen compound biosynthetic process	−0.32	−1.55	0.00
	GO translational initiation	−0.38	−1.55	0.01
	GO gland development	−0.75	−1.54	0.01
	GO intracellular transport	−0.35	−1.52	0.01
	HP abnormal nervous system electrophysiology	−0.60	−1.52	0.03
	GO intracellular protein transport	−0.35	−1.48	0.02

*textit| NES| > 1, and p-value < 0.05 was considered significant.*

### Protein–Protein Interaction Network and Critical Sub-Network Construction

**FIGURE 4:**
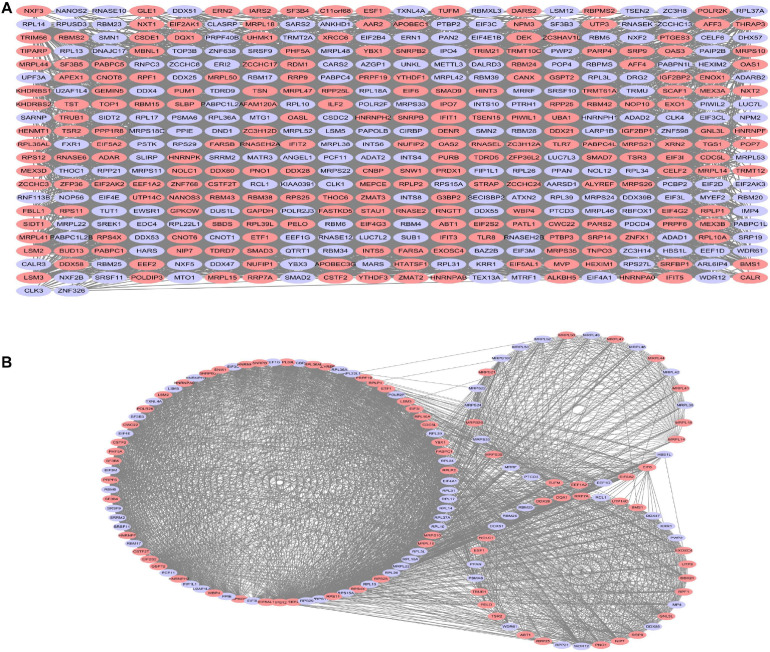
Protein–protein interaction (PPI) network analysis **(A)** and critical sub-network analysis **(B)**. Lilac ovals, downregulated RBPs; pink ovals, upregulated RBPs.

All DE-RBPs except disconnected nodes were imported in the STRING database to construct the PPI network including 422 PPI nodes and 5,840 edges, which were then visualized with cytoscape in Figure 4A. Then, critical sub-networks were separated out to construct sub-networks by MCODE as shown in Figure 4B.

### Prognostic Risk Model in Pancreatic Adenocarcinoma

**FIGURE 5:**
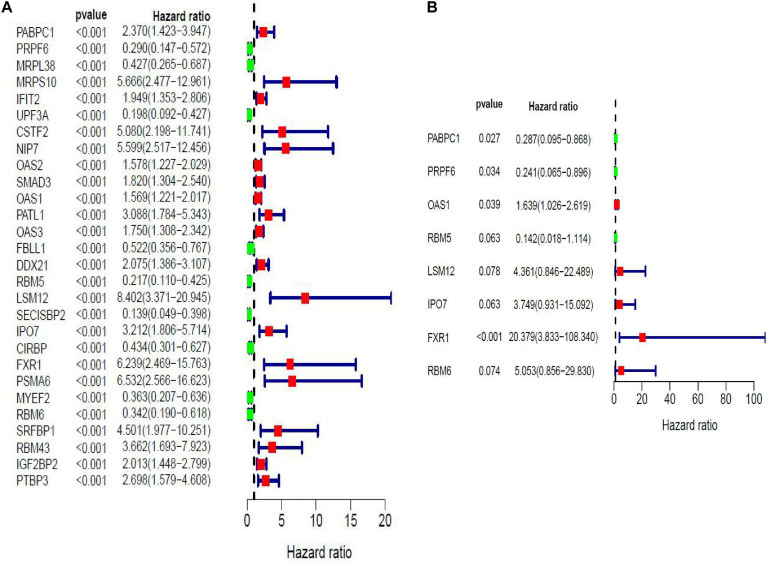
Forest maps of prognosis-related RBPs (pRBPs) in PAAD by univariate **(A)** and multivariate **(B)** Cox regression analysis. Green boxes, lower risk RBPs with hazard ratio (HR) < 1; red boxes, higher risk RBPs with HR > 1.

**FIGURE 6:**
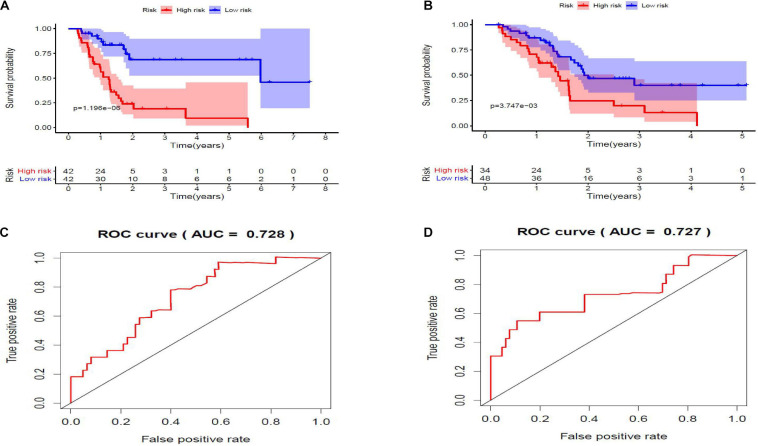
**(A,B)** Kaplan–Meier survival curves. Low-risk score subgroup and high-risk score subgroup are highlighted by blue curve and red curve, respectively. The cutoff value of risk score is determined by its median value. Panels **(A,B)** are modeling cohort (*p* = 1.196e-06) and validation cohort (*p* = 3.747e-03), respectively. **(C,D)** Receiver operating characteristic (ROC) curves. *X*- and *Y*-axes represent false-positive rate and true-positive rate, respectively. Area under curve (AUC) value of modeling cohort and validation cohort are 0.728 and 0.727, respectively.

**FIGURE 7:**
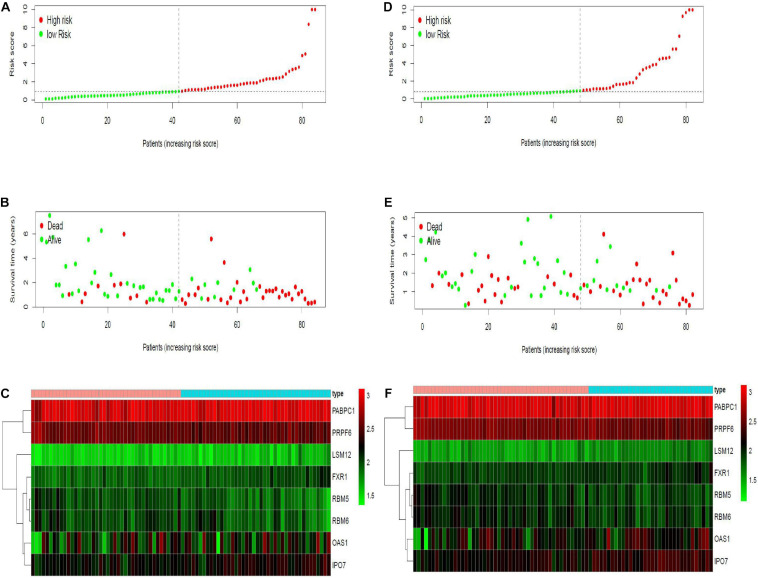
Risk curves in modeling and validation cohort. *X*- axes of the six figures all represent PAAD samples, arranged in order of increasing risk score. **(A,D)** Dot plot of risk score. *Y*-axis represents risk score. Red and green color dots represent, respectively, high- and low-risk score samples. **(B,E)** Dot plot of survival. *Y*-axis represents survival times (years). Red and green color dots represent, respectively, dead and living PAAD samples. **(C,F)** Heat map. *Y*-axis represents eight modeling RBPs. Higher, medium, and lower expression levels of RBPs are shown in red, black, and green, respectively. Blue and pink bars on top of the heat map indicate low- and high-risk score samples, respectively.

**FIGURE 8:**
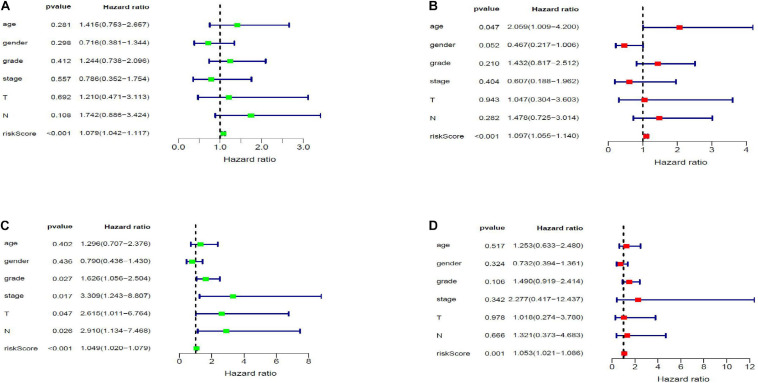
Forest map of the relationship of risk score and clinical characteristics with prognosis in PAAD by univariate **(A,C)** and multivariate **(B,D)** Cox analysis. Panels **(A,B)** and **(C,D)** are forest maps of modeling and validation cohort, respectively.

**FIGURE 9:**
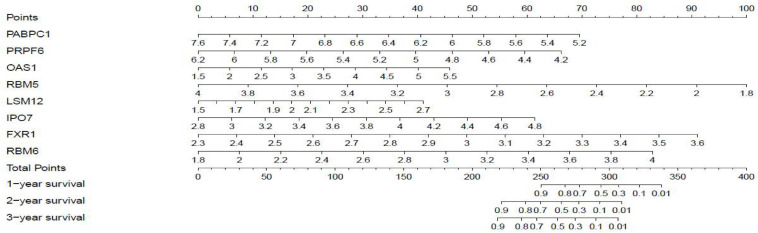
Nomogram for predicting 1-, 3-, and 5-year overall survival (OS) of PAAD patients based on modeling cohort.

Twenty eight prognosis-related RBPs (pRBPs) were selected by univariate Cox regression (Figure 5A). It should be noted that patients in TCGA were classified into two cohorts randomly and equally, one cohort for modeling and the other for validating. Multivariate Cox regression was further applied to select pRBPs to construct a prognostic risk model in modeling cohort. These eight pRBPs are shown in Table 3 and Figure 5B. Of the eight pRBPs, three pRBPs (PABPC1, PRPF6, and RBM5) were low risk and five pRBPs (OAS1, LSM12, IPO7, FXR1, and RBM6) were high risk. According to coefficient values of modeling pRBPs and corresponding pRBP expression value, risk scores of PAAD patients in modeling cohort and validation cohort were calculated. The formulation was: risk score = (−1.2467 × ExpPABPC1) + (−1.4246 × ExpPRPF6) + (0.4943 × ExpOAS1) + (−1.9552 × ExpRBM5) + (1.4727 × ExpLSM12) + (1.3216 × ExpIPO7) + (3.0145 × ExpFXR1) + (1.6201 × ExpRBM6). Kaplan–Meier survival curves indicated that higher risk score was especially relevant to poor prognosis (*p* = 1.196e-06); the validation cohort got the same conclusion (*p* = 3.747e-03) (Figures 6A,B). ROC curves verified favorable accuracy (AUC value was 0.728 and 0.727, respectively) (Figures 6C,D). Risk curves further confirmed that the higher the risk score, the lower the survival rate, and the worse prognosis (Figure 7). Then we analyzed the effect of risk score on the prognosis of PAAD by univariate and multivariate Cox regression. As shown in Table 4 and Figure 8, high risk score is an independent worse prognostic indicator for PAAD patients either in the modeling cohort or in the validation cohort. We then established a nomogram by assigning to quantize clinical factors of PAAD patients such as age, gender, grader, stage, tumor size, lymph gland involvement, and risk score (Figure 9). Based on multivariate Cox regression analysis and the influence of various clinical characteristics on the prognosis, each value level of each factor was scored. By adding up each score, the total score was obtained, and the total score can evaluate 1-, 3-, and 5-year survival rate, which might assist clinicians in making clinical decisions for PAAD patients.

**TABLE 3:** RNA-binding proteins (RBPs) selected to construct prognostic risk model by multivariate Cox regression analysis in modeling cohort.

**RBPs**	**Coef**	**HR**	**95% CI**	***p*-value**
PABPC1	−1.2467	0.2874	0.0952–0.8683	0.0271
PRPF6	−1.4246	0.2406	0.0646–0.8957	0.0337
OAS1	0.4943	1.6393	1.0260–2.6192	0.0387
RBM5	−1.9552	0.1415	0.0180–1.1142	0.0633
LSM12	1.4727	4.3609	0.8456–22.4889	0.0785
IPO7	1.3216	3.7493	0.9315–15.0916	0.0629
FXR1	3.0145	20.3788	3.8333–108.3398	0.0004
RBM6	1.6201	5.0533	0.8561–29.8298	0.0737

*Coef, coefficient value; HR, hazard ratio; CI, confidence interval.*

**TABLE 4:** Effects of clinical factors and risk score on prognosis of PAAD patients analyzed by univariate and multivariate Cox analysis.

	**Clinical factors**	**Univariate Cox**			**Multivariate Cox**		
		**HR**	**95% CI**	***P*-value**	**HR**	**95% CI**	***P*-value**
Modeling cohort	Age	1.4147	0.7532–2.6574	0.2807	2.0586	1.0091–4.1999	**0.0472**
	Gender	0.7157	0.3812–1.3440	0.2982	0.4671	0.2168–1.0064	0.0519
	Grade	1.2443	0.7385–2.0965	0.4116	1.4324	0.8168–2.5120	0.2099
	Stage	0.7860	0.3522–1.7544	0.5567	0.6072	0.1880–1.9618	0.4044
	*T*	1.2105	0.4707–3.1128	0.6919	1.0465	0.3040–3.6033	0.9425
	*N*	1.7419	0.8861–3.4241	0.1075	1.4781	0.7249–3.0139 1.0547–1.1403	0.2825
	Risk Score	1.0789	1.0424–1.1167	**0.0000**	1.0967		**0.0000**
Validation cohort	Age	1.2958	0.7067–2.3759	0.4021	1.2531	0.6333–2.4796	0.5170
	Gender	0.7898	0.4361–1.4301	0.4359	0.7325	0.3943–1.3607	0.3245
	Grade	1.6263	1.0562–2.5041	**0.0272**	1.4897	0.9193–2.4140	0.1056
	Stage	3.3088	1.2432–8.8068	**0.0166**	2.2772	0.4170–12.4372	0.3421
	*T*	2.6153	1.0112–6.7644	**0.0474**	1.0182	0.2743–3.7796	0.9784
	*N*	2.9102	1.1341–7.4682	**0.0263**	1.3213	0.3728–4.6832	0.6661
	Risk Score	1.0491	1.0197–1.0793	**0.0010**	1.0529	1.0210–1.0857	**0.0010**

*HR, hazard ratio; CI, confidence interval; P < 0.05 was statistically significant, and P-value less than 0.05 in the table are shown in red bold.*

### Expression, Survival Value and Gene Set Enrichment Analysis of 8 Modeling RNA-Binding Proteins

**FIGURE 10:**
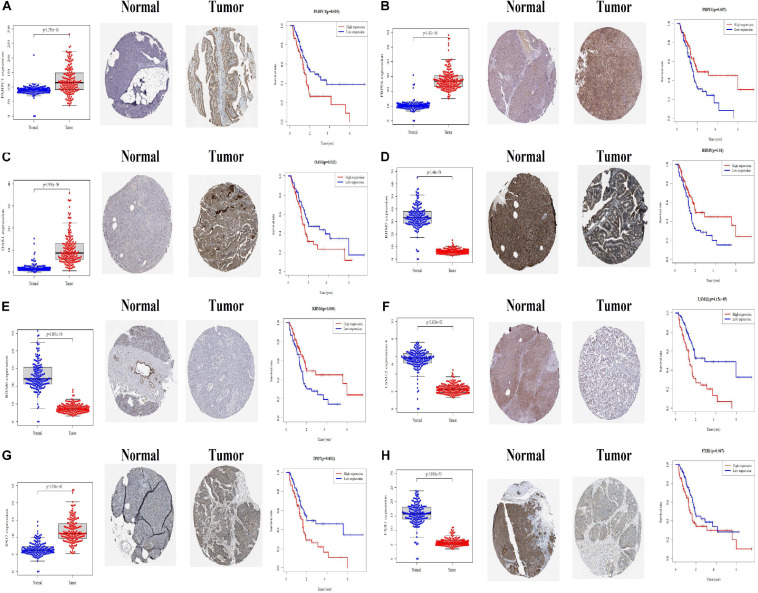
Differential expression and Kaplan–Meier survival curves of eight modeling RBPs in PAAD based on TCGA, HPA, and KM-PLOT databases. **(A)** PABPC1; **(B)** PRPF6; **(C)** OAS1; **(D)** RBM5; **(E)** RBM6; **(F)** LSM12; **(G)** IPO7; **(H)** FXR1. *P* < 0.05 is considered significantly.

**FIGURE 11:**
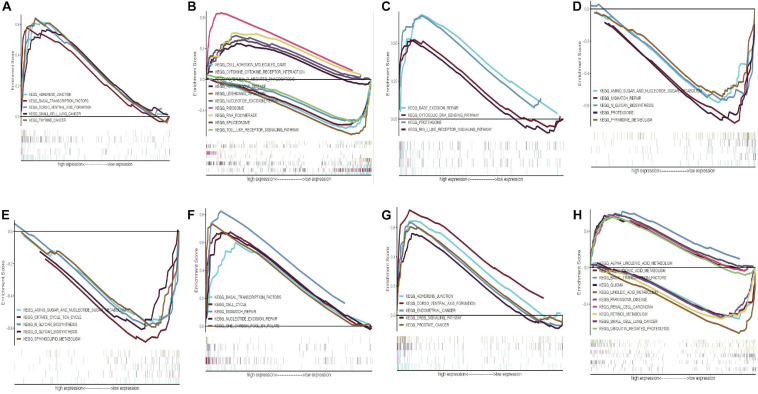
Gene set enrichment analysis of eight modeling RBPs in PAAD. **(A)** PABPC1; **(B)** PRPF6; **(C)** OAS1; **(D)** RBM5; **(E)** RBM6; **(F)** LSM12; **(G)** IPO7; **(H)** FXR1. Only the top 5 enrichment pathways according to NES in each phenotype were displayed if available. NOM *P*-value < 0.05 and FDR *q*-value < 0.25 were considered significant. NES, normalized enrichment score.

We further analyzed the expression differences of eight modeling RBPs between normal and cancer cases from RNA and protein expression level. The results are shown in Figure 10. Of the eight modeling RBPs, half were upregulated including PABPC1, PRPF6, OAS1, IPO7, and half were downregulated including RBM5, RBM6, LSM12, and FXR7 in cancer cases. Results of immunohistochemical staining are in accordance with RNA expression level. KM survival curves are shown in Figure 10. All of the 8 modeling RBPs were associated with overall survival in PAAD. Higher expression levels of PABPC1, OAS1, LSM12, IPO7, and FXR1 predicted poor prognosis, and adversely, higher expression levels of PRPF6, RBM5, and RBM6 predicted favorable prognosis. To gain insight into the molecular mechanisms in which these RBPS may be involved, we performed GSEA for these modeling RBPs (Figure 11 and Supplementary Table 1). In Supplementary Table 1, we listed all significant pathways and separated the top 5 in sheet 2. According to NES of GSEA, signaling pathways that were enriched in highly expressed phenotypes in PABPC were thyroid cancer, adherens junction, dorso ventral axis formation, small cell lung cancer, and basal transcription factors; signaling pathways that were enriched in highly expressed phenotypes in PRPF6 were ribosome, spliceosome, nucleotide excision repair, RNA polymerase, Huntington’s disease, etc.; signaling pathways that were enriched in low expressed phenotypes in PRPF6 were cytokine–cytokine receptor interaction, cell adhesion molecule cams, leishmania infection, Toll-like receptor signaling pathway, FC gamma R-mediated phagocytosis, etc.; signaling pathways that were enriched in highly expressed phenotypes in OAS1 were rig I-like receptor signaling pathway, base excision repair, proteasome, and cytosolic DNA-sensing pathway; signaling pathways that were enriched in low expressed phenotypes in RBM5 were amino sugar, and nucleotide sugar metabolism, mismatch repair, pyrimidine metabolism, proteasome, *N*-glycan biosynthesis, etc.; signaling pathways that were enriched in low expressed phenotypes in RBM6 were sphingolipid metabolism, citrate cycle (TCA cycle), *O*-glycan biosynthesis, amino sugar and nucleotide sugar metabolism, *N*-glycan biosynthesis, etc.; signaling pathways that were enriched in highly expressed phenotypes in LSM12 were cell cycle, mismatch repair, nucleotide excision repair, one carbon pool by folate, basal transcription factors, etc.; signaling pathways that were enriched in highly expressed phenotypes in IPO7 were dorso-ventral axis formation, adherence junction, endometrial cancer, ERBB signaling pathway, prostate cancer, etc.; signaling pathways that were enriched in highly expressed phenotypes in FXR1 were ubiquitin-mediated proteolysis, basal transcription factors, small cell lung cancer, renal cell carcinoma, glioma, etc.; signaling pathways that were enriched in low expressed phenotypes in FXR1 were linoleic acid metabolism, arachidonic acid metabolism, alpha linolenic acid metabolism, retinol metabolism, Parkinson’s disease, etc.

## Discussion

Pancreatic adenocarcinoma is the most malignant digestive tract tumor with a 5-year survival rate of less than 10% (McGuigan et al., 2018). In the background of rapid development of diagnosis and treatment for malignant tumors, the worse status of early diagnosis and treatment of PAAD has not been greatly improved. Recently, several studies have revealed that parts of RBPs significantly influence the progression of PAAD. For example, MSL1 and MSL2 were drivers of PAAD, which were proved to promote the transition of pancreatic intraepithelial neoplasias to PAAD and to increase the aggression of PAAD (Fox et al., 2016; Kudinov et al., 2017). Considering the significance of RBPs in PAAD, in this study, we systematically explored RBP expression in PAAD in order to provide potential biomarkers for diagnosis and treatment of PAAD.

First, we screened DE-RBPs and pRBPs in PAAD. RBPs (453) are expressed differentially between normal and PAAD samples, 28 of which are prognosis related. Of the 453 DE-RBPs, most of them are functionally associated with ribosome, ribonuclease, and spliceosome. Ribonucleases (RNases) are a group of hydrolytic enzymes to catalyze RNA molecule degradation, which are classified by two types: endoribonucleases and exoribonucleases (Shlyakhovenko, 2016). Biologically, RNases take part in many physiological activity such as RNA metabolism, remobilization of phosphate, defensin-like activity, and senescence (Fang et al., 2012; Tatsuta et al., 2014). Researchers were attracted by the cytotoxic effects (inducing apoptosis) of RNases, which can be applied in anticancer activity. One study indicated that defection of RNases inhibited the apoptosis of prostate cancer cells (Malathi et al., 2004). The loss of RNase T2 stimulated ovarian tumorigenesis (Acquati et al., 2013). Ribosome is one of the important organelles of protein synthesis. Studies have shown that in order to satisfy the tumor cells’ continuous growing, it is necessary to increase ribosome biogenesis to maintain high protein synthesis efficiency. Therefore, abnormal ribosome biogenesis may result in the occurrence of carcinoma (Pelletier et al., 2018). Specific ribosomal proteins were found to be upregulated in a variety of tumors. For example, the expression of RPL5, RPS3, RPS6, RPS8, and RPS12 in colorectal cancer was higher than that in normal colorectal mucosa. The expression of RPL15 was upregulated in gastric cancer tissues and cell lines. The mRNA level of RPS8, RPL12, RPL23A, RPL27, and RPL30 was detected as upregulated in hepatocellular carcinoma tissues and cell lines (Wang et al., 2015). Related mechanism studies show that ribosome biosynthesis is an important part of Ras/Raf/MEK/ERK, MYC, and PI3K/Akt/mTOR pathways, which are proven to drive malignant tumors. Besides, therapeutic effects of many anticancer drugs used in the clinic are partly by destroying ribosome biosynthesis. For example, cisplatin, oxaliplatin, adriamycin, and mitomycin C inhibit ribosome synthesis at the rRNA transcription level, while 5-fluorouracil-camptothecin inhibits ribosome formation at the rRNA processing level (Burger and Eick, 2013; Derenzini et al., 2018). There is no doubt that spliceosome is a hot spot in the field of cancer research in recent years. The spliceosome consists of five snRNP containing U1, U2, U4, U5, U6, and several splicing factors (SFs) (Hsu et al., 2015). The disorder of SFs expression can activate tumor-related alternative splicing events, leading to cell carcinogenesis ultimately (David and Manley, 2010; Zhang and Manley, 2013). Then, the KEGG pathway enrichment analysis indicated that these DE-RBPs are mainly enriched in RNA transport, ribosome, mRNA surveillance pathway, spliceosome, ribosome biogenesis in eukaryotes, RNA degradation, influenza A, as well as aminoacyl-tRNA biosynthesis, which echoed GO functional analysis.

In addition, we established a PPI network with 422 nodes and 5,840 edges for DE-RBPs. Of 28 pRBPs, 8 RBPs including PABPC1, PRPF6, OAS1, RBM5, LSM12, IPO7, FXR1, and RBM6 are selected to construct a prognostic risk model by multivariate Cox regression. PABPC1 (poly A binding protein, cytoplasmic 1) is known to participate in RNA degradation and translation (Takashima et al., 2006). PABPC1 promotes growth and progression of gastric cancer cells by regulating miR-34c and induces proliferation of hepatocellular carcinoma cells by promoting entry into the S phase (Zhang et al., 2015; Zhu et al., 2015). On the contrary, PABPC-1 is considered as a tumor suppressor in glioblastoma cells by binding to BDNF-AS (Su et al., 2020). PRPF6 is reported to be related with spliceosome in colon cancer (Adler et al., 2014) and androgen receptor (AR) signaling in hepatocellular carcinoma (HCC) (Song et al., 2020). Most studies about OAS1 are limited in bioinformatics analysis. A study from Robert et al. indicated that OAS1 expression was correlated with azacytidine (AZA) sensitivity in the NCI-60 tumor cell lines and was a biomarker for predicting AZA sensitivity of tumor cells (Banerjee et al., 2019). Besides, one gene expression profiling combining bioinformatics analysis in regard to PAAD identified that OAS1 was related to worse prognosis of PAAD (Tang et al., 2019). A basic experiment showed that pancreatic cancer cell lines with high OAS expression were resistant to oncolytic virus therapy (Moerdyk-Schauwecker et al., 2013). RBM5 (RNA-binding motif protein 5) and RBM6 (RNA-binding motif protein 6) were initially reported as tumor suppressors. Both of them map to the 3p21.3 region with frequent alteration in lung cancer (Lerman and Minna, 2000; Oh et al., 2002). RBM5 was observed to promote cell apoptosis and retard tumor growth. RBM5 was highly downregulated in breast cancer (Rintala-Maki et al., 2004) and prostate cancer (Zhao et al., 2012). By contrast, some studies indicated that RBM5 was upregulated in breast cancer and ovarian cancer as a result of overexpression of oncogene EGFR-2 (Zhang et al., 2019). It seems that both overexpression and downexpression of RBM5 influence the progression of cancer. RBM6 mRNA was reported to be highly upregulated in many cancer types, such as breast cancer, malignant fibrous histiocytoma, ovary cystadenoma, non-Hodgkin’s lymphoma, and pancreatic cancer. A study from Chen Huang et Al. proved that upregulated RBM6 in pancreatic cancer may be released into the blood, which could be a candidate and potential serum biomarker for early diagnosis of pancreatic cancer (Duan et al., 2019). Elevated IPO7 was found in various cancer types such as colorectal cancer (CRC), which can be explained by the fact that the transcription of gene IPO7 was suppressed by p53 and promoted by c-Myc (Golomb et al., 2012), as well as glioma, in which IPO7 increased promoter activity of FOXM1, leading to the nuclear import of GL1 and glioma development (Xue et al., 2015). IPO7 was found to be upregulated in several pancreatic cancer lines (Suit2 and MIA PaCa2) (Cui et al., 2017). A lot of studies suggested the tumor-promotive function of FXR1, and FXR1 was highly upregulated in oral squamous cell carcinoma (Majumder et al., 2016), lung squamous cell carcinoma (Comtesse et al., 2007), head and neck squamous cell carcinoma (Qie et al., 2017), triple negative breast carcinoma (Qian et al., 2017), ovarian carcinoma (Zhao et al., 2017), etc. It not only has potential diagnostic and prognostic value but also can predict specific metastasis and response to chemoradiotherapy. The function of LSM12 has not been reported in the development of cancer. Our analysis suggests that LSM12 may be a high-risk RBP with carcinogenesis in PAAD. The abovementioned researches and our analysis of ROC curve, validation cohort, risk curves, as well as nomogram proved the reliability and accuracy of the model.

In general, we successfully constructed an RBP-associated prognostic risk model in PAAD, which has potential clinical application prospect. However, there are still many limitations in our study. First, we did not compare the prognostic risk model with other recognized prognostic factors such as KRAS and TP53. Second, most of the eight modeling RBPs are not reported in PAAD, and functional studies are needed to verify the roles of modeling RBPs in PAAD.

## Conclusion

We constructed a prognostic risk model consisting of eight hub RBPs in PAAD based on TCGA database. The corresponding results not only help us understand the significant values of RBPs in occurrence and progression of PAAD but also help develop new therapeutic targets and prognostic molecular markers.

## Data Availability Statement

The original contributions generated for this study are included in the article/Supplementary Material, further inquiries can be directed to the corresponding author.

## Ethics Statement

Ethical review and approval was not required for the study on human participants in accordance with the local legislation and institutional requirements. Written informed consent for participation was not required for this study in accordance with the national legislation and the institutional requirements.

## Author Contributions

XW: data download and analysis and manuscript writing. ZS: manuscript writing and modification. SC: manuscript arrangement and manuscript writing. WW, YW, JJ, and QM: reviewing literature. LZ: manuscript guidance. All authors contributed to the article and approved the submitted version.

## Conflict of Interest

The authors declare that the research was conducted in the absence of any commercial or financial relationships that could be construed as a potential conflict of interest.

## Footnotes

1 https://xenabrowser.net/datapages/

2 https://portal.gdc.cancer.gov/

3 http://www.string-db.org/

4 https://www.proteinatlas.org/

## Supplementary Material

Supplementary Figure 1 Gene set enrichment analysis (GSEA) analysis for differentially expressed RBPs between normal and tumor groups in PAAD. *P*-value < 0.5 and | NES| > 1were considered significantly.

Supplementary Table 1 Gene set enrichment analysis (GSEA) for eight modeling RBPs. (| NES| > 1 and NOM *p*-value < 0.05 were considered significantly).
