# Supplementary material for: Construction of an RNA-Binding Protein-Related Prognostic Model for Pancreatic Adenocarcinoma Based on TCGA and GTEx Databases
Source: Front Genet. 2021 Jan 27;11:610350. doi: 10.3389/fgene.2020.610350 (PMC7873872; doi:10.3389/fgene.2020.610350)
Supplement: Supplementary Table 1 — Gene set enrichment analysis (GSEA) for eight modeling RBPs. (| NES| > 1 and NOM p-value < 0.05 were considered significantly). [file Image_1.pdf]

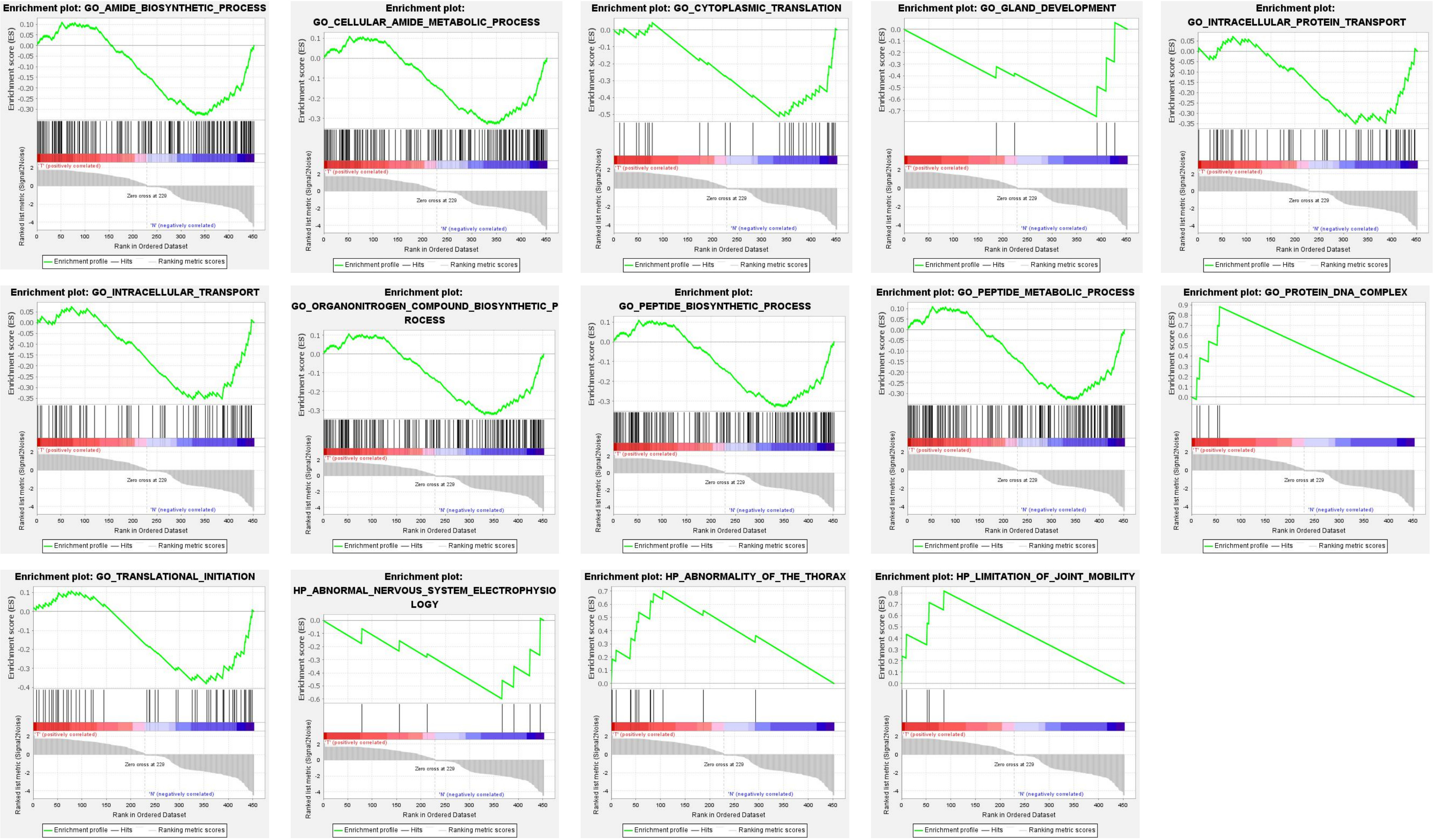

Supplementary Figure 1 GSEA analysis for differentially expressed RBPs between normal and tumor groups in PAAD. P-value<0.05 and |NES|>1were considered significantly.
